# Supplementary material for: Disproportionate declines of formerly abundant species underlie insect loss
Source: Nature. 2023 Dec 20;628(8007):359–64. doi: 10.1038/s41586-023-06861-4 (PMC11006610; doi:10.1038/s41586-023-06861-4)
Supplement: Supplementary file 1 — This file contains Supplementary Methods (Search terms used in all languages) and the metadata for Supplementary Data 1–3 (available at https://doi.org/10.5281/zenodo.10115304). [file 41586_2023_6861_MOESM1_ESM.pdf]

nature portfolio

https://doi.org/10.1038/s41586-023-06861-4

Supplementary information

Disproportionate declines of formerly abundant species underlie insect loss

In the format provided by the authors and unedited

Supplementary methods 1

Search strings

English (www.webofscience.com)

ALL=(("insect" OR "arthropod" OR "invertebrate" OR “beetle*” OR “butterfl*” OR “moth*” OR “flies” OR “bee*” OR “grasshopper” OR “herbivore” OR “pollinator” OR “mosquito*”) AND (“biomass” OR "abundance" OR "community dynamics" OR “temporal” OR “trend” OR “monitor*” OR “dynamics” OR “richness” OR “composition”) NOT (“Marine”) NOT (“Malaria”))

Refined by “long”

Russian (www.elibrary.ru)

(бентос, мезофауна, насекомые, зоопланктон, жесткокрылые, двукрылые, перепончатокрылые, коллембола, чешуекрылые, прямокрылые) + (динамика, многолетний)

Spanish (www.scielo.org)

(also covering literature in Portugese, since in this database a Spanish abstract is always provided):

("insecto" OR "insectos" OR "artropodo" OR "artropodos" OR "invertebrado" OR "invertebrados" OR "escarabajo" OR "escarbajos" OR "cucarron" OR "cucarrones" OR "mariposa" OR "mariposas" OR "polilla" OR "polillas" OR "moscas" OR "mosca" OR "mosquitos" OR "mosquito" OR "zancudo" OR "zancudos" OR "abeja" OR "abejas" OR "abejorro" OR "abejorros" OR "saltamonte" "saltamontes" OR "grillo" OR "grillos" OR "cucarachas" OR "cucaracha" OR "cigarras" OR "chicharras" OR "cigarra" OR "chicharra" OR "libelulas" OR "hormigas" OR "herbivoro" OR "herbivoros" OR "polinizador" OR "polinizadores" OR "Coleoptera" OR "Diptera" OR "Lepidoptera" OR "Hymenoptera" OR "Odonata" OR "Formicidae" OR "Plecoptera" OR "Trichoptera" OR "Ephemeroptera") AND ("biomasa" OR "abundancia" OR "dinamica" OR "temporal" OR "tendencia" OR "monitoreo" OR "riqueza" OR "composicion" OR "diversidad") AND NOT ("malaria" OR "marinos")

Chinese

SU=(’昆虫‘+’节肢动物’+’无脊椎动物’+’龙虱’+’甲虫’+’鞘翅目’+’蝴蝶’+’蛾’+’蝇’+’蜂’+’蝗虫’+’草食动物’+’传粉*’+’蚊’+’蠓’+’鞘翅目’+’双翅目’+’鳞翅目’+’膜翅目’+’蜻蜓目’+’蚁科’+’襀翅目’ +’毛翅目’+’蜉蝣目’) AND SU=(‘群落动态’+’时间’+’趋势’+’监测’+’动态’+’丰富度’+’组成’+’多样性’) NOT SU=('海洋'+'疟疾')

Metadata Supplementary Data 1.

Rds file (for R) with the complete data frame from which all analyses can be reproduced. Code for analysis is available on github: https://github.com/roelvanklink/final-insect-diversity-trends1

Column explanation:

Column name	Explanation
Plot_ID	Unique identifier for each site. Always nested in a Datasource_ID
Datasource_ID	Unique identifier for each dataset. ID's from BioTIME and GPDD were left as is.
Datasource_name	Name of Datasource_ID, for ease of recognition. Redundant with Datasource_ID.
Year	Year of sampling event
Period	Time of the year of sampling event (month / season)
Metric	Metric of assemblage size. Factor:<br>- *Abundance*: total number of individuals observed per sampling event<br>- *Richness*: total number of taxa observed per sampling event<br>- *rarefiedRichness*: number of species expected to be observed per sampling event had the minimum observed number of individuals in any year been found<br>- *CoverageRichness.8:* Coverage based richness: Expected number of species if 80% of the community had been sampled<br>- *Shannon*: raw Shannon -Wiener entropy (not yet converted to its Effective Number of Species)<br>- *ENSPIE*: inverse Simpson index $1/\sum p_i^2$<br>- *logNr020*: number of species in SAD interval 0 – 20%<br>- *logNr2040*: number of species in SAD interval 20 – 40%<br>- *logNr4060*: number of species in SAD interval 40 – 60%<br>- *logNr6080*: number of species in SAD interval 60 – 80%<br>- *logNr80100*: number of species in SAD interval 80 – 100%<br>- *logNrQ1*: Number of species in first SAD quartile (See Fig. S1)<br>- *logNrQ2*: Number of species in second SAD quartile (See Fig. S1)<br>- *logNrQ3*: Number of species in third SAD quartile (See Fig. S1)<br>- *logNrQ4*: Number of species in fourth SAD quartile (See Fig. S1)

Number	Count of insects by any metric observed at this sampling event
Realm	Realm of sampling: here, always Terrestrial
Continent	Continent at which sampling took place
Region	Grouping of Countries or states into geographical units providing sufficient data for analysis
Country	Nation state in which sampling took place
Country_State	Country or state in which sampling took place (finer scale than Country)
Location	Unique identifier of study area or reserve of the sampling event. Not always nested in Dataset.
cYear	variable Year centered around mean for Inla analysis
iYear	Index year for random intercept
rYear	Copy of iYear for random slope
rYear2	Copy of iYear for random slope
Period_4INLA	Unique number for each Period for random intercept in Inla analysis
Plot_ID_4INLA	Unique number for each Plot_ID for random intercept in Inla analysis
Datasource_ID_4INLA	Unique number for each Datasource_ID for random intercept in Inla analysis
Location_4INLA	Unique number for each Location for random intercept in Inla analysis
Plot_ID_4INLAs	Unique number for each Plot_ID for random slope in Inla analysis
Datasource_ID_4INLAs	Unique number for each Datasource_ID for random slope in Inla analysis
Location_4INLAs	Unique number for each Location for random slope in Inla analysis

Metadata Supplementary Data 2.

Datasource_ID	Unique identifier for each dataset. ID's from BioTIME and GPDD were left as is.
Plot_ID	Unique identifier for each site. Always nested in a Datasource_ID
Location	Unique identifier of study area or reserve of the sampling event. Not always nested in Dataset.
Realm	Realm of sampling: here, always Terrestrial
Year	Year of sampling event
Number	Number of individuals (or mean number of individuals over iterations) of this taxon
Taxon	Taxon name as provided in original data source
Level	Taxonomic level (species, genus, family etc)
Rank	Numerical variable for taxonomic level
Phylum	Phylum
Class	Class
Subclass	Subclass
Order	Order
Suborder	Suborder
Family	Family
Subfamily	Subfamily
Genus	Genus
Species	Specific epithet
validTaxon	Incompletely harmonized and updated taxonomic names: Genus + species
Note	Notes or descriptions from original datasets
cYear	variable Year centered around mean for Inla analysis
CGYr1.0	Initial abundance group, based on year 1 only
CG1.2	Initial abundance group, based on years 1 and 2
CG1.5	Initial abundance group, based on years 1 – 5
CGallYrs	Abundance group, based on all years
iYear	Index year for random intercept
rYear	Copy of iYear for random slope
rYear2	Copy of iYear for random slope
Datasource_ID_4INLA	Unique number for each Datasource_ID for random intercept in Inla analysis
Plot_ID_4INLA	Unique number for each Plot_ID for random intercept in Inla analysis
Location_4INLA	Unique number for each Location for random intercept in Inla analysis
Plot_ID_4INLAs	Unique number for each Plot_ID for random slope in Inla analysis
Datasource_ID_4INLAs	Unique number for each Datasource_ID for random slope in Inla analysis
Location_4INLAs	Unique number for each Location for random slope in Inla analysis

TaxonPlot_4INLA	Unique number for each within a plot for random intercept in Inla analysis
TaxonPlot_4INLAs	Unique number for each within a plot for random slope in Inla analysis

Metadata Supplementary Data 3.

Datasource_ID	Unique identifier for each dataset. ID’s from BioTIME and GPDD were left as is.
Datasource_name	Name for easier identification of datasets
Location	Unique identifier of study area or reserve of the sampling event. Not always nested in Dataset.
Plot_ID	Unique identifier for each site. Always nested in a Datasource_ID
Plot_name	Unique plot name
TaxonPlot_4INLAs	Unique identifier for population (a species in a site)
CGYr1.0	Initial abundance group in year 1
Class	Taxonomic class
Order	Taxonomic order
Taxon	Taxon name as provided in original data source
Level	Taxonomic level
slope	Mean trend slope of the population, calculated as the model random slope per population + the mean random slopes at the study, location and site levels + correction factor for RtM
CImin	Minimum credible interval of trend, calculated the model's lower 90% CI of the population level random slope + the lower 90% CI's of the random slopes at the study, location and site levels + the fixed year effect + correction factor for RtM
CImax	Maximum credible interval of trend of trend, calculated the model's upper 90% CI of the population level random slope + the upper 90% CI's of the random slopes at the study, location and site levels + the fixed year effect + correction factor for RtM
percchange	Calculated percentage change per year for the trend slope
Population trend	Qualification whether there is at least weak evidence (zero outside the 80% CI of the trend slope):<br>no trend = 80% credible interval overlaps 0<br>>80% certain positive: 0 < than the lower 80% CI<br>>80% certain negative: 0 > than the upper 80% CI
