# Supplementary material for: Disproportionate declines of formerly abundant species underlie insect loss
Source: Nature. 2023 Dec 20;628(8007):359–64. doi: 10.1038/s41586-023-06861-4 (PMC11006610; doi:10.1038/s41586-023-06861-4)
Supplement: Supplementary file 2 — Reporting Summary [file 41586_2023_6861_MOESM2_ESM.pdf]

nature portfolio

Corresponding author(s): Roel van Klink

Last updated by author(s): Aug 20, 2023

Reporting Summary

Nature Portfolio wishes to improve the reproducibility of the work that we publish. This form provides structure for consistency and transparency in reporting. For further information on Nature Portfolio policies, see our Editorial Policies and the Editorial Policy Checklist.

Statistics

For all statistical analyses, confirm that the following items are present in the figure legend, table legend, main text, or Methods section.

n/a	Confirmed	
☐	☒	The exact sample size (*n*) for each experimental group/condition, given as a discrete number and unit of measurement
☐	☒	A statement on whether measurements were taken from distinct samples or whether the same sample was measured repeatedly
☐	☒	The statistical test(s) used AND whether they are one- or two-sided<br>*Only common tests should be described solely by name; describe more complex techniques in the Methods section.*
☐	☒	A description of all covariates tested
☐	☒	A description of any assumptions or corrections, such as tests of normality and adjustment for multiple comparisons
☐	☒	A full description of the statistical parameters including central tendency (e.g. means) or other basic estimates (e.g. regression coefficient) AND variation (e.g. standard deviation) or associated estimates of uncertainty (e.g. confidence intervals)
☐	☒	For null hypothesis testing, the test statistic (e.g. *F*, *t*, *r*) with confidence intervals, effect sizes, degrees of freedom and *P* value noted<br>*Give P values as exact values whenever suitable.*
☐	☒	For Bayesian analysis, information on the choice of priors and Markov chain Monte Carlo settings
☐	☒	For hierarchical and complex designs, identification of the appropriate level for tests and full reporting of outcomes
☐	☒	Estimates of effect sizes (e.g. Cohen's *d*, Pearson's *r*), indicating how they were calculated

Our web collection on statistics for biologists contains articles on many of the points above.

Software and code

Policy information about availability of computer code

Data collection	Some data were digitized using ImageJ software, and some were digitized using the MetaDigitise package for R. All data were processed in R between 2018 and 2023
Data analysis	We used R 4.2.2 for all data processing , and the INLA package for statistical analyses. Visualization was done using the GGplot2 package. We used hierarchical Bayesian modeling to estimate the temporal slope of the various biodiversity metrics (i.e. the explanatory variable w as 'Year'

For manuscripts utilizing custom algorithms or software that are central to the research but not yet described in published literature, software must be made available to editors and reviewers. We strongly encourage code deposition in a community repository (e.g. GitHub). See the Nature Portfolio guidelines for submitting code & software for further information.

Data

Policy information about availability of data

All manuscripts must include a data availability statement. This statement should provide the following information, where applicable:

- Accession codes, unique identifiers, or web links for publicly available datasets
- A description of any restrictions on data availability
- For clinical datasets or third party data, please ensure that the statement adheres to our policy

All Data as analyses in this paper are available as supplementary material, and the underlying raw data are available on Knowledge Network for Biocomplexity. The

code used for analysis is available at Zenodo: https://doi.org/10.5281/zenodo.8369189

Research involving human participants, their data, or biological material

Policy information about studies with human participants or human data. See also policy information about sex, gender (identity/presentation), and sexual orientation and race, ethnicity and racism.

Reporting on sex and gender	NA
Reporting on race, ethnicity, or other socially relevant groupings	NA
Population characteristics	NA
Recruitment	NA
Ethics oversight	NA

Note that full information on the approval of the study protocol must also be provided in the manuscript.

Field-specific reporting

Please select the one below that is the best fit for your research. If you are not sure, read the appropriate sections before making your selection.

☐ Life sciences ☐ Behavioural & social sciences ☒ Ecological, evolutionary & environmental sciences

For a reference copy of the document with all sections, see nature.com/documents/nr-reporting-summary-flat.pdf

Ecological, evolutionary & environmental sciences study design

All studies must disclose on these points even when the disclosure is negative.

Study description	We evaluated the biodiversity change of assemblages of terrestrial insects, arachnids and Entognatha (springtails and allies) over time to understand the patterns underlying insect declines. We performed this study by means of synthesis of existing and openly accessible data.
Research sample	Our aim was to evaluate as many datasets as were available under the following restrictions: We collected time series of insect/ arachnid/Entognatha assemblages (at least all individuals of a family or higher taxonomic level, counted), assessed using consistent methodology with at least 9 years between the first and last sampling year, that were or could be made openly accessible. A low proportion of non-target taxa - other arthropods such as crustaceans and myriapods(<10%) was acceptable, whenever these could not be separated from the target taxa.) Our sample size was thus dependent on the amount of openly available. We used 106 datasets. The exact provenance of each study is detailed in Extended Data table 5.<br>The sample population is the world, but we are aware that our data are biased in various ways. Therefore, the data and analysis mostly represent those locations from which data are available. We show the biases in several sensitivity analyses
Sampling strategy	The sample size was determined by the availability of data. When necessary and possible, we used rarefaction methods to equalize sampling effort across years. Experimental treatments (where researchers actively manipulated environmental conditions) were excluded.
Data collection	Standardized and non-standardized data searches were performed by Roel van Klink, Jonathan Chase and Konstantin Gongalsky. Data were digitzied, extracted and standardized by Roel van Klink, Minghua Shen, Abdul Al-Hemiary and Nina Naderi. The collection of the original data involved numerous data collectors and data analysts.
Timing and spatial scale	Data searches were performed from 2018 - 2021, but the collated data go back to 1951,Data from around the world were included, but there is a bias towards Europe and North America (detailed in the paper)
Data exclusions	No data exclusions other than data or years that did not fit our analysis criteria (mostly in cases of taxonomic or methodological inconsistencies).
Reproducibility	All code and analytical data for statistical tests is available on Zenodo https://doi.org/10.5281/zenodo.8369189. All raw data can be found at KNB: https://knb.ecoinformatics.org/view/urn%3Auuid%3Ab338c276-1d3f-4cc7-a192-cad846083455
Randomization	NA (no treatments were analysed)
Blinding	NA

Did the study involve field work? ☐ Yes ☒ No

Reporting for specific materials, systems and methods

We require information from authors about some types of materials, experimental systems and methods used in many studies. Here, indicate whether each material, system or method listed is relevant to your study. If you are not sure if a list item applies to your research, read the appropriate section before selecting a response.

Materials & experimental systems

n/a	Involved in the study
☒	☐ Antibodies
☒	☐ Eukaryotic cell lines
☒	☐ Palaeontology and archaeology
☒	☐ Animals and other organisms
☒	☐ Clinical data
☒	☐ Dual use research of concern
☒	☐ Plants

Methods

n/a	Involved in the study
☒	☐ ChIP-seq
☒	☐ Flow cytometry
☒	☐ MRI-based neuroimaging
