# Supplementary material for: Disproportionate declines of formerly abundant species underlie insect loss
Source: Nature. 2023 Dec 20;628(8007):359–64. doi: 10.1038/s41586-023-06861-4 (PMC11006610; doi:10.1038/s41586-023-06861-4)
Supplement: Supplementary file 3 — This file contains Supplementary Tables 1–5. [file 41586_2023_6861_MOESM3_ESM.pdf]

Supplementary Table 1| Data availability of terrestrial insects for various biodiversity metrics. Discrepancies in data availability between metrics are caused by the possibility of calculating each metric on the available data. For example, some time series may be extremely sparse (have only few species or individuals), and some metrics can only be calculated on integers, which were mostly, but not always available. Abundance and richness data were taken from original publications, also when no underlying data were available.

Metric	Studies	Sites
Abundance	105	920
Richness	75	780
Diversity	57	551
Rarefied richness	53	529
Coverage based richness	51	528
SAD intervals	57	551
Populations	56	584

Supplementary Table 2| Characteristics of the full dataset at the study level. The number of taxa is only applicable to datasets reporting full community composition.

	Minimum	Median	Maximum
Number of sites	1	3	138
Study duration	9	20	64
Number of years with data	2	11	53
Start year	1951	1990	2009
End year	1985	2011	2021
Number of taxa	2	56.5	1757

Supplementary Table 3 | Number of datasets available per continent and biodiversity metric.

Metric	Africa	Asia	Europe	Latin America	North America	Oceania
Abundance	1	13	54	14	18	5
Richness	0	8	39	10	15	3
Community composition	0	4	27	9	15	2

Supplementary Table 4 | Details on the source data underlying this study.

Datasource ID	Taxon	Place	Metrics	Start	End	Time span	Number of years data	Number of sites	Number of species	url	License	Reference
79	Butterflies	United Kingdom	ARC	1976	1985	10	10	11	39		c	58
249	Beetles and moths	Denmark	ARC	1994	2009	16	16	1	1462	url	d	59
294	Butterflies	Vietnam	ARC	2004	2013	10	5	4	210		c	47
301	Grasshoppers	USA: Kansas	ARC	1982	2018	37	31	15	45	url	c	47
313	Grasshoppers	USA: Minnesota	ARC	1989	2006	18	16	19	58	url	c	47
375	Beetles	Japan	ARC	2005	2014	10	10	22	357		c	47
380	Butterflies	United Kingdom	ARC	1978	1987	10	10	1	26		a	47
465	Moths	Czech republic	ARC	1967	1992	26	23	1	133		a	46
502	Aphids	United Kingdom	ARC	1969	1990	22	22	16	32		a	46
1006	Moths	United Kingdom	ARC	1992	2015	24	24	13	452	url	b	60
1102	Ground beetles	Netherlands	ARC	1959	2019	61	53	32	172		c	22
1261	Ants	USA: Massachusetts	ARC	2003	2018	16	14	4	21	url	c	61
1263	Butterflies	United Kingdom	ARC	1993	2015	23	23	13	44	url	b	62
1266	Froghoppers	United Kingdom	ARC	1993	2015	23	23	9	2	url	b	63
1267	Ground beetles	United Kingdom	ARC	1993	2015	23	23	47	112	url	b	64
1310	Plant- and leafhopppers	Panama	ARC	1974	1993	20	20	1	1757		a	65
1312	Moths	Czech republic	AR	1963	1991	29	29	3	-		a	66
1319	Grasshoppers	USA: New Mexico	ARC	1992	2013	22	22	4	56	url	c	67
1324	Spiders, Ground beetles	Netherlands	ARC	1969	2008	40	39	4	211		a	68
1328	Hoverflies	United Kingdom	ARC	1972	2001	30	30	1	95		c	22
1335	Ladybeetles	Czech republic	ARC	1983	2010	28	2	3	13		a	69
1339	Grasshoppers	USA: Montana	ARC	1986	2016	31	30	4	12	url	a	70
1340	Moths	Hungary	ARC	1962	2009	48	46	7	878	url	d	71
1346	Ants	USA: New Mexico	ARC	1995	2005	11	11	1	5	url	c	72
1349	Arthropods	USA: Arizona	ARC	2006	2019	14	14	23	494	url	c	73

1361	Grasshoppers	USA: Georgia	ARC	2000	2016	17	17	5	5	url	c	[74]
1365	Ground beetles	USA: Minnesota	A	1980	2005	26	2	3	-	url	a	[75]
1367	Ground beetles	Italy	ARC	1980	2009	30	4	6	17		a	[76]
1377	Orchidbees	Panama	ARC	1980	2000	21	18	1	35		a	[77]
1378	Butterflies	Costa Rica	A	2003	2012	10	10	1	-		a	[78]
1379	Butterflies	Ecuador	A	1994	2004	11	11	1	-		a	[79]
1382	Terrestrial arthropods	Chile	A	2003	2014	12	12	1	-		a	[80]
1384	Moths	New Zealand	AR	1962	1988	27	2	1	-		a	[81]
1385	Dung beetles and Carrion beetles	Brazil: Amazonas	A	1986	2000	15	2	1	-		a	[82]
1391	Soil macroinvertebr ates	Russia: Murmansk	AR	1986	2010	25	3	7	-		a	[83]
1392	Butterflies	Slovakia	ARC	2001	2012	12	6	1	57		a	[84]
1393	Terrestrial insects	Russia: Tatarstan	A	1978	1995	18	18	3	-		a	[85]
1394	Butterflies	Uganda	ARr	2000	2011	12	12	1	-		a	[86]
1396	Springtails	Germany	ARC	1980	2000	21	9	1	-		a	[87]
1397	Ants	Hungary	A	1981	2017	37	28	1	-		a	[88]
1398	Beetles	Czech republic	ARC	1975	2007	33	7	3	301		a	[89]
1400	Ground beetles	Russia: Buryatia	A	1988	2007	20	20	4	-		a	[90]
1401	Beetles	Russia: Lipetsk Oblast	AR	1995	2004	10	10	1	-		a	[91]
1403	Parasitoid wasps	Russia: Sverdlovsk Oblast	A	1994	2003	10	4	1	-		a	[92]
1404	Arthropods	Greenland	ARC	1996	2019	24	23	7	76	url	h	[93]
1405	Saproxylic beetles	Finland	AR	1990	1999	10	10	2	-		a	[94]
1406	Arthropods	Russia: Orenburg Oblast	A	1990	2004	15	9	1	-		a	[95]
1407	Ground beetles	Russia: Tver Oblast	A	1996	2012	17	17	2	-		a	[96]
1411	Soil fauna	Austria	A	1998	2012	15	2	4	-		a	[97]
1413	Soil fauna	South Korea	AR	1998	2007	10	10	8	-		a	[98]
1416	Caterpillars	USA: New Hampshire	A	1986	1997	12	11	3	-	url	h	[99]
1419	Ants	Australia: Western Australia	ARC	1980	1997	18	2	3	47		a	[100]
1434	Spiders	Switzerland	A	1994	2004	11	11	1	-		a	[101]
1445	Ants	USA: Arizona	ARC	1977	2009	33	28	2	21		d	[102]
1446	Ground beetles	Russia: Kursk Oblast	A	1983	1999	17	17	3	-		a	[103]
1458	Springtails	Russia: Novgorod Oblast	AR	1981	1995	15	5	3	-		a	[104]

1459	Ground beetles	Russia: Moscow Oblast	ARC	1974	1990	17	6	2	24		a	105
1460	Rove beetles	Russia: Leningrad Oblast	AR	1983	2005	23	4	1	-		a	106
1461	Hoverflies	Russia: Khabarovsk Krai	A	1988	2013	26	5	1	-		a	107
1462	Butterflies	Russia: Primorsky Krai	A	1986	2005	20	20	1	-		a	108
1464	Parasitoid wasps	Belarus	AR	1990	2000	11	11	1	-		a	109
1465	Moths	Malaysia	AR	1965	2007	43	2	5	-		a	110
1467	Hymenoptera	Belarus	A	1986	2003	18	16	1	-		a	111
1468	Bumblebees	Spain	ARC	1988	2008	21	2	1	24		a	112
1470	Cuckoo wasps	Belarus	A	1985	2005	21	21	1	-		a	113
1471	Beetles	Ukraine	A	2003	2011	9	9	1	-		a	114
1472	Moths	Hungary	AR	1990	2004	15	8	1	-		a	115
1474	Grasshoppers bugs, plant- and leafhoppers	Germany	ARC	1951	2009	59	2	9	196		c	116
1475	Plant and leafhoppers	Germany	ARC	1963	2010	48	7	27	179		c	117
1476	Butterflies	USA: Wisconsin	ARC	1990	2020	31	31	53	86		a	18
1477	Butterflies	USA: Wisconsin	ARC	1988	2021	34	33	45	121		a	18
1478	Butterflies	USA: Wisconsin	ARC	1990	2021	32	32	35	101		a	18
1479	Arthropods	Australia: Tasmania	A	1999	2015	17	9	6	-		c	18
1480	Arthropods	Australia: Tasmania	A	2001	2012	12	4	14	-		c	18
1481	Butterflies	Israel	A	2009	2018	10	10	10	-		c	118
1487	Arthropods	Puerto Rico	ARC	1991	2016	26	15	30	167		a	119
1493	Flying insects	United Kingdom	A	2000	2009	10	10	1	-		a	120
1494	Sawflies	United Kingdom	A	1970	1988	19	19	5	-		a	121
1496	Flying insects	United Kingdom	A	1972	1998	27	27	1	-		a	122
1497	Ladybeetles	United Kingdom	A	2006	2016	11	11	4	-		a	123
1501	Butterflies	Ireland	ARC	1992	2016	25	16	2	25	url	c	124
1502	Ground beetles	China: Hebei	ARC	1997	2014	18	2	3	44		a	125
1505	Mining and galling insects	Finland	ARC	2003	2013	11	11	1	21		d	126, 127
1512	Ground beetles	Germany	Rr	1994	2017	24	24	1	-		a	128
1515	Dung beetles	Spain	ARC	1983	2017	35	2	1	50		a	129
1516	Saproxylic beetles	Sweden	AR	2001	2013	13	3	2	-		a	130

1518	Butterflies	USA: Ohio	A	1996	2016	21	21	58	-		c	18, 131
1521	Springtails	Denmark	AR	1985	1999	15	8	2	-		a	132
1524	Flying insects	Netherlands	A	1997	2017	21	21	1	-		a	133
1528	Moths	Finland	AR	1993	2012	20	20	54	-	url	d	134, 135
1530	Ants	Brazil: Amazonas	AR	2006	2014	9	2	1	-		a	136
1531	Dung beetles	Brazil: Mato Grosso do Sul	ARC	1990	2015	26	26	1	40		a	137
1533	Bees	Brazil: Parana	ARC	1955	2018	64	2	15	28		a	138
1535	Bees	Brazil: Sao Paulo	ARC	1990	2018	29	3	1	11		a	139
1539	Beetles	USA: New Hampshire	ARC	1973	2017	45	8	3	42		c	140
1541	Butterflies	Panama	ARC	2009	2017	9	9	1	288		d	141, 142
1543	Flying insects	China: Shandong	ARD	2003	2017	15	15	1	-		a	143
1544	Ants	Australia: Queensland	ARC	1992	2013	22	19	10	99	url	d	144, 145
1547	Moths	South Korea	ARC	2005	2017	13	13	6	1038	url	c	146, 147
1548	Ground beetles	Finland	ARC	1995	2006	12	5	3	21		a	148
1549	Moths	United Kingdom	ARC	1993	2013	21	21	1	373		a	149
1550	Spiders, beetles, grasshoppers, true bugs, plant- and leafhoppers	Germany	AR	2008	2017	10	10	138	-		e	5
1558	Calyptrate flies	China: Hubei	ARD	2006	2016	11	11	4	-		a	150
1560	Bees	Brazil: Parana	ARC	1981	2015	35	2	1	154		a	151
1561	Bees	Brazil: Parana	ARC	1962	2004	43	3	1	55		a	152
1562	Aphids	USA: US Midwest	ARC	2006	2019	14	14	33	81		d	153

Open access licenses:

a. PD: public domain (all data extracted from papers)
b. OGL: Open Government License (UK)
c. CC-BY
d. CC0
e. CC-BY-NC
f. CC-BY-ND
g. ODC: Open Data Commons
h. no shar: data openly accessible, but no redistribution of data or derived products is allowed

More detailed metadata are available in the data repository on the Knowledge Network Biocomplexity[20] and in[16].

Metrics: A: Abundance, R: Richness, C: Community composition and evenness, Rr: Rarefied richness, D = Diversity (Shannon / Simpson)

Supplementary Table 5 | Correction factors for regression-to-the-mean, calculated as the difference between the mean slope estimate of all available years, and the dataset censored by the first or the first three years.

Initial abundance interval	1-year censoring	3-year censoring
<20%	-0.00136	-0.00339
20-40%	0.00372	0.00977
40-60%	0.01065	0.01863
60-80%	0.01366	0.01968
80-100%	0.01251	0.01829
